# Immune Activities in Choroids of Visually Impaired Smyth Chickens With Autoimmune Vitiligo

**DOI:** 10.3389/fmed.2022.846100

**Published:** 2022-04-25

**Authors:** Jordan Sorrick, Wilson Huett, Kristen A. Byrne, Gisela F. Erf

**Affiliations:** ^1^Department of Biological Sciences, University of Arkansas, Fayetteville, AR, United States; ^2^Department of Poultry Science, University of Arkansas System Division of Agriculture, Fayetteville, AR, United States

**Keywords:** uveitis, vitiligo-associated diseases, choroid melanocytes, Smyth chicken vitiligo model, autoimmune vitiligo

## Abstract

Vitiligo is a common dermatological disorder affecting 1–2% of the world’s population. It is characterized by postnatal, autoimmune destructions of melanocytes in the skin, resulting in patches of depigmentation. Autoimmunity in vitiligo may also affect melanocytes in non-integumental tissues, including the eyes where choroidal melanocytes are the target of the autoimmune response. The Smyth line (SL) of chicken is the only animal model that spontaneously and predictably develops all clinical and biological manifestations of autoimmune vitiligo. In SL vitiligo (SLV), destruction of epidermal melanocytes in growing feathers (GFs) involves a melanocyte-specific, Th1-mediated cellular immune response. Smyth chickens may also exhibit uveitis and vision impairment. Previous studies established a strong association between SLV and vision impairment, including similar pathology in affected eyes and GFs. To determine the presence, types, and activities of choroid infiltrating mononuclear cells, we collected eyes before, near onset, and during active SLV from sighted, partially blind, and blind SL chickens. All SL chickens with vision impairment had SLV. Immunohistochemistry and quantitative reverse transcriptase-PCR analyses revealed mononuclear cell and cytokine expression profiles in the autoimmune destruction of melanocytes in choroids that are identical to those described in GF, demonstrating the systemic nature of autoimmunity against melanocytes in SLV. In addition, we observed aberrant melanogenesis in SL eyes. The immunopathogenesis in SL vision impairment resembles human vitiligo-associated ocular diseases, especially Vogt–Koyanagi–Harada syndrome and sympathetic ophthalmia. Hence, the Smyth chicken autoimmune vitiligo model provides the opportunity to expand our understanding of spontaneous autoimmune pigmentation disorders and to develop effective treatment strategies.

## Introduction

Vitiligo is a progressive pigmentary disorder characterized by autoimmune loss of epidermal melanocytes in the skin, resulting in white patches of the skin. Vitiligo affects about 1% of the world’s population (1). Other autoimmune diseases associated with this skin disease include autoimmune thyroiditis, alopecia areata, and uveitis (2–4). The Smyth line (SL) chicken, previously known as the delayed amelanosis (DAM) chicken, is the only animal model that spontaneously and predictably develops all aspects of autoimmune vitiligo, including the associated autoimmune diseases observed in humans (5–10). Since its development in the 1970s by poultry geneticist J. Robert Smyth, Jr. at the University of Massachusetts, Amherst, early SL characterization focused on the vitiligo-like depigmentation in growing feathers (GFs) and on the visual impairment which were observed in 85 and 40% of SL chickens, respectively (5, 6, 11).

The time course, incidence, and pathology of SLV development and the tight link between visual impairment and SLV persisted through decades of selections and is still present in the only remaining SL subline (SL101) maintained by G. F. Erf at the University of Arkansas Systems, Division of Agriculture in Fayetteville. The Arkansas SL vitiligo model was established in 1996 from fertilized eggs provided by Dr. J. R. Smyth, Jr. and consists of three lines: the vitiligo-susceptible SL with an 80–95% vitiligo-incidence, the vitiligo-susceptible parental Brown line (BL) which continues to have a 1–2% vitiligo incidence, and the vitiligo-resistant Light-brown Leghorn (LBL) which serves as the pigmentation control. All three lines are homozygous for MHC (*B* locus) 101. The incidence of visual problems in the SLV populations varies year to year from 5 to 20%, although mating of affected birds greatly increases the prevalence of visual problems in offspring (9, 10).

Examination of pigment cell changes and tissue pathology as vitiligo and visual impairment developed revealed similar disease progression in GF and eyes (6, 11, 12–14). SL chicks hatched with functioning pigment cells in the down plumage, choroid, and retinal pigment epithelium. In most cases, SL vitiligo (SLV) developed between 4.5 and 12 weeks of age. Visual problems occurred 1–3 weeks after vitiligo onset and were never observed in birds without SLV. Histological and ultrastructural examination of GF from 2- to 3-week-old chicks revealed that epidermal melanocytes in the barb ridge synthesized abnormal melanosomes with irregularly shaped surfaces and pigmented extension. The earliest signs of impending vitiligo development were retraction of melanocyte dendrites, clumping of pigment, intracellular compartmentalization of melanosomes, reduction of pigment transfer to keratinocytes (barbule cells), and, eventually, melanocyte degeneration. Melanocyte degeneration was accompanied by the appearance of small (lymphocytes) and large (macrophages) mononuclear leukocytes in the pulp of GF, cessation of melanocyte migration into the barb ridge, and absence of pigmentation. Similarly, changes in choroidal melanocytes included swelling of cell bodies and dendrites, dendrite retractions, melanosome irregularities, and intracellular compartmentalization. Subsequent melanocyte degeneration also was associated with mononuclear cell infiltration in the choroid. Extensive mononuclear leukocyte infiltration was associated with damage to the retinal pigment epithelium (RPE), first observed in the peripapillary region of the optic nerve and pecten. In blind birds, pathological changes in the RPE involved the entire retina, while in partially blind chicks only choroids were affected with no or minimal RPE abnormalities (6, 7, 11, 12–14).

Research by Erf and coworkers on the immune system’s role in SLV development included characterization of pulp-infiltrating mononuclear cell populations by immunohistochemistry, fluorescence-based flow cytometry, and gene-expression analysis at the transcriptome level. For this, the GF target tissue was collected repeatedly from the same birds before and throughout the development and progression of SLV, and data were aligned with respect to individual SLV onset. Briefly, the mononuclear leukocytes infiltrate consisted of T cells, namely, CD4+, CD8+, αβ TCR+, and γδ TCR+ T cell subsets, B cells, and macrophages. Collectively, T cells (90% αβ TCR+) infiltrated the pulp of GF 3–4 weeks before SLV onset, reached the highest levels near SLV onset, remained elevated throughout active SLV (4–8 weeks), and returned to near baseline levels when melanocyte destruction was complete. Within the T cell populations, CD4+ T helper cells reached peak levels 3–7 days before SLV onset and started to decline 3–7 days after onset, whereas CD8+ cytotoxic T cells reached maximal levels within days after onset and remained at high levels until complete loss of melanocytes. At the onset of SLV, the ratio between CD4+ and CD8+ T cells in the feather pulp was near 1.0 and decreased to below 0.4 as melanocyte destruction progressed (15–17). During active SLV, CD8+ T cells were observed in close physical association with apoptotic epidermal melanocytes (18). T cell infiltration was accompanied by a sharp and short-lived increase in B cells (Bu-1+ and IgM+) 3 days before SLV onset. B cell levels peaked 3–7 days after onset and declined rapidly thereafter. Macrophage levels were consistently elevated during active SLV (16, 17). Furthermore, the melanocyte-specific autoimmune response was shown to be a T helper type 1 (Th1), interferon (IFN)-γ polarized cell-mediated response. IFN-γ expression paralleled the T cell infiltration profiles and MHC class II staining and was accompanied by high expression of IL-21 and IL-10, and elevated expression of IL-1, IL-6, and IL-8 (CXCL8) (17, 19, 20).

Considering the established link between vitiligo development and vision problems in SL chickens, and the reported similarities in melanocytes degeneration and mononuclear cell infiltration in affected GF and eyes, we hypothesize that autoimmune activities in the eye parallel those observed in vitiliginous GF. The objective of this study was to examine the presence, type, and activities of mononuclear cells in the eyes of fully sighted, partially blind, and blind SL chickens and in normally sighted BL and LBL controls. For this, eyes were collected at 1 week of age when no abnormalities were reported in previous studies, at 4 weeks when early signs of melanocyte degenerative processes were described, and at 12 weeks when melanocyte degeneration, mononuclear cells infiltration, and vision problems were observed in vitiliginous SL chickens. To gain insight into melanocyte activities in eye tissue of SL and controls, the expression of melanogenesis-related genes also was examined.

## Materials and Methods

### Experimental Animals

All procedures involving animals were approved by the Institutional Animal Care and Use Committee (protocol # 08011). For this study, SL, BL, and LBL chicks from a large pedigreed breeder replacement hatch were raised under conventional husbandry conditions by G. F. Erf on the Agricultural Experiment Station’s Poultry Farm in Fayetteville, AR, United States. The SL chicken has a high incidence of vitiligo development (about 80–95%), and frequently shows signs of visual impairment (5–20%). Non-vitiliginous BL (parental line; <2% incidence of vitiligo, no vision impairment) and LBL (vitiligo resistant) chickens were also included as normally sighted controls.

### Experimental Approach and Tissue Collection

Chickens were checked weekly for visual acuity based on their response to hand signals as described in Boissy et al. (11). During weekly visual acuity tests, birds also were checked for vitiligo development by evaluating pigmentation loss in the bottom portion (newest growth) of GFs (15). At 1, 4, and 12 weeks of age, three normally sighted (NV) chickens from each of the LBL, BL, and SL populations were randomly selected for tissue collection. In addition, eyes were collected from SL chickens with impaired vision (SL-IV), including one 4-week-old, four 8-week-old, and three 12-week-old SL with partial vision, and three 12-week-old blind SL chickens. All SL-IV chickens had vitiligo, including the one 4-week-old chick identified as having partial vision loss.

For eye collection, chicks were euthanized by CO_2_ inhalation (1 and 4 weeks) or i.v. injection of sodium pentobarbital (8 and 12 weeks). Both eyes were collected from each bird, placed in separate aluminum cups in OCT freezing medium with the pupil and cornea facing the side of the cup. Eye samples were snap-frozen in liquid nitrogen and stored at -80°C until processed for immunohistochemical staining and gene-expression analyses.

### Immunohistochemical Staining

Using a cryostat (Microm Microtome HM505 E) at −29°C, thick, horizontal sections (100 μm) were cut from one eye of each bird until nearing maximal eye diameter and the optic nerve and pecten were visible (middle of the eye). The cutting thickness was then changed to 7 μm to cut a total of 8 sections for immunohistochemical (IHC) staining. Individual sections were placed on poly-L-lysine coated microscope slides and fixed in acetone for 5 min. Eye sections were blocked overnight with 10% horse serum in PBS in a humidifying chamber. The next day, slides were stained as described in Erf et al. (15). Briefly, sections were incubated with primary mouse-anti-chicken monoclonal antibodies (Southern Biotech, Birmingham, AL, United States) specific for CD4, CD8, TCR1 (γδ T cells), Bu-1 (B cells), IgM, KUL01 (macrophages), or MHC class II. The binding of primary antibodies (all mouse IgG1) was detected using biotinylated horse-antimouse IgG secondary antibody (Vektor Laboratories Inc, Burlingham, CA, United States) followed by the addition of streptavidin and biotin-conjugated peroxidase mixture (Vektastain Elite reagents, Vector Laboratories, Inc) and activated 3,3-diaminobenzidine tetrahydrochloride (DAB) substrate (DAB; Sigma Chemical Company, Saint Louis, MO, United States). To determine nonspecific staining, a mouse IgG1 isotype control antibody (Southern Biotech) was used in place of the primary antibody on one section of each sample. Sections were counterstained with Dako Mayer’s Hematoxylin stain (Dako North America, Inc., Carpinteria, CA, United States) and covered with mounting medium (Aquamount; Lerner Laboratories, Pittsburgh, PA, United States) and glass coverslips.

### Tissue Analysis

Sections were viewed at 100× magnification using a bright-field Olympus BX50 microscope (Olympus America Inc., Center Valley, PA, United States) equipped with a Cool SNAP digital camera and computer. The isotype control-stained sections were evaluated for nonspecific staining, endogenous peroxidase activity, choroidal melanocyte damage (scores 1–3, intact melanocytes to clumps of pigment), and appearance of the RPE (scores 1–3, intact to damaged/destroyed RPE). The relative amount (% area) of immunostaining for various leukocyte populations and MHC class II+ cells in choroids was determined by image analysis using ImagePro^®^ Plus software (Media Cybernetics, Inc., Rockville, MD, United States) as described in Erf and Ramachandran (21).

### Targeted Gene-Expression Analysis by Quantitative Reverse Transcriptase PCR

Using the other eye, eye tissue was collected from ten, 70-μm-thick frozen, horizontal sections cut from the center of each eye. The eye tissue was pulled out of each section, avoiding the vitreous humor portion, and placed in a microfuge tube. RNA isolation was carried out using the materials and procedures described in the RNeasy mini kit (Qiagen Inc., Valencia, CA, United States). Quantitative and qualitative RNA analysis was conducted using the lab-on-chip Experion™ RNA StdSens analysis kits and the Experion™ analyzer (Bio-Rad, Hercules, CA, United States). The isolated RNA was reverse transcribed into complementary DNA (cDNA) using Taqman reverse transcription reagents (Applied Biosystems, Foster City, GA, United States). cDNA was used in a quantitative real-time polymerase reaction (qPCR) assay (17). Target gene primers and probes for chicken cytokines interleukin-1β (IL-1β), IL-6, IL-8/CXCL8, IL-10, IL-21, and interferon-γ (IFN-γ), melanogenesis-related genes MC1R (melanocortin 1-receptor), MITF (melanocyte-inducing transcription factor), TYR (tyrosinase), TRP1 (tyrosinase-related protein-1), and TRP2 (dopachrome tautomerase) (Table 1). cDNA from a single LBL with normal sight served as the calibrator sample and the 28S subunit of ribosomal RNA as the endogenous control. The ΔΔ*CT* method was used to determine relative gene expression (fold change) (17).

**TABLE 1:** Primer and probe sequences for relative expression analysis of chicken target genes.

Target	Primer/probe	Sequences (5′to 3′)	Accession NO.
28S	Forward	GGCGAAGCCAGAGGAAACT	X59733
	Reverse	GACGACCGATTTGCACGTC	
	Probe	AGGACCGCTACGGACCTCCACCA	
IL-1β	Forward	GCTCTACATGTCGTGTGTGATGAG	AJ245728
	Reverse	TGTCGATGTCCCGCATGA	
	Probe	CCACACTGCAGCTGGAGGAAGCC	
IL-6	Forward	GCTCGCCGGCTTCGA	AJ250838
	Reverse	GGTAGGTCTGAAAGGCGAACAG	
	Probe	AGGAGAAATGCCTGACGAAGCTCTCCA	
IL-8	Forward	GCCCTCCTCCTGGTTTCA	AJ009800
	Reverse	TGGCACCGCAGCTCATT	
	Probe	TCTTTACCAGCGTCCTACCTTGCGACA	
IL-10	Forward	CATGCTGCTGGGCCTGAA	AJ621614
	Reverse	CGTCTCCTTGATCTGCTTGATG	
	Probe	CGACGATGCGGCGCTGTCA	
IL-21	Forward	GTGGTGAAAGATAAGGATGTCGAA	NM_001024835.1
	Reverse	TGCCATTCTGGAAGCAGGTT	
	Probe	TGCTGCATACACCAGAAAACCCTGGG	
IFN-γ	Forward	GTGAAGAAGGTGAAAGATATCATGGA	Y07922
	Reverse	GCTTTGCGCTGGATTCTCA	
	Probe	TGGCCAAGCTCCCGATGAACGA	
Tyr	Forward	ATAATGCCCTTCACATCTACATGAAT	NM_204160.1
	Reverse	GCTCAAAAATGCTGTCAACAAATG	
	Probe	CTCAATGTCCCAAGTACAAGGCTCTGCG	
Trp1	Forward	GGAACCATTTGTAACAGCACTGAAG	NM_205045.1
	Reverse	CCATAGGCCGTGCAACATTT	
	Probe	CGGTCCCATCCGTAGAAATCCTGCTG	
Trp2	Forward	CCTTTCCCGGCATGAGTTT	NM_204935.1
	Reverse	AGCGCATTCCTGAAGCTGAA	
	Probe	CAGTCCTCCGTTTTTCCGCAATTCCA	
Mitf	Forward	AAGAACTGGGCACCTTGATACC	NM_205029.1
	Reverse	GATGTAGTCCACTGATGCTTTTAGAATAG	
	Probe	AAATCAAACGACCCGGATATGCGCTG	
Mc1r	Forward	GCCCTTCTTCTTCCACCTCAT	NM_001031462
	Reverse	AGAGGTTGAAATAGCTGAAGAAGCA	
	Probe	CTCATCGTCACCTGCCCCACCAAC	

*Primers and probes were designed using Primer Express 3.0 (Applied Biosystems, Foster City, CA, United States). Primer and probe oligos were synthesized by MWG Biotech, High Point, NC, United States.*

### Statistical Analysis

One-way ANOVA was conducted to compare data between groups of chickens (LBL-NV, BL-NV, and SL-NV, SL-IV partial, and SL-IV-blind at each age). ANOVA was followed by Fisher’s least-significant difference (LSD) multiple means comparison to determine differences between the individual groups. There were no differences between BL and LBL eyes at any age, hence, data were pooled (controls) for comparison with SL. Similarly, there were no differences in SL-IV partial samples collected at 8 and 12 weeks for any parameter examined, and, except for melanocyte and RPE scores, between SL-IV partial and SL-IV blind birds. Therefore, for all data, except melanocyte and RPE scores, 12-week comparisons were made between controls, SL-NV, and SL-IV, with SL-IV including all data from partially sighted (8 and 12 weeks) and blind (12 weeks) samples. Statistical analyses were carried out using SYSTAT software (SYSTAT Software Inc., Chicago, IL, United States). All data are reported as mean ± SEM and means are considered different if *P* ≤ 0.05.

## Results

### Histology and Leukocyte Infiltration Profiles

**FIGURE 1:**
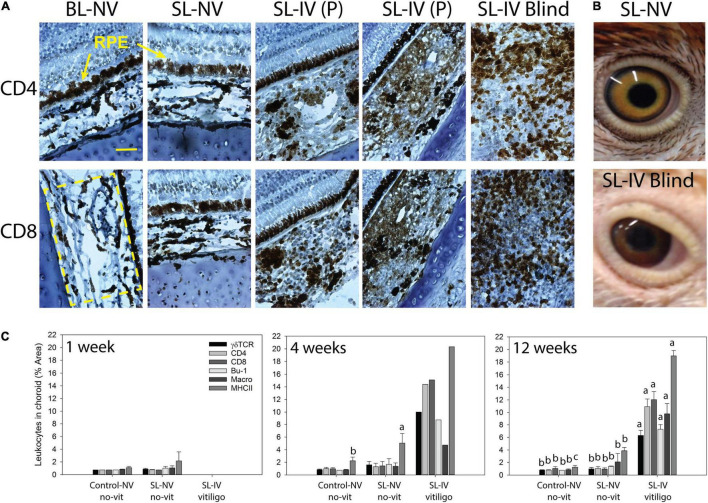
Mononuclear leukocyte presence and MHC class II expression in choroids of healthy controls and Smyth line (SL) chickens with and without vitiligo and visual impairment. **(A)** Representative CD4 or CD8 IHC-stained cells in frozen sections (7 μm) of eyes from 12-week-old chickens. BL-NV: Normally sighted (NV) parental Brown line (BL) control without vitiligo (no-vit); SL-NV: normally sighted, vitiligo-prone Smyth line (SL) chicken without vitiligo; SL-IV: vision-impaired SL chickens [partially sighted (P) or blind], all SL-IV chickens had vitiligo. RPE (retinal pigmented epithelium) is destroyed in blind SL-IV chickens. **(B)** The appearance of eyes in an SL-NV chicken without vitiligo (top) and a vitiliginous, blind SL-IV chicken (bottom). **(C)** Presence (% area) of T cell subsets (γδ, CD4, and CD8), B cells, macrophages, and MHC class II+ cells in choroids of controls, SL-NV, and SL-IV (includes P and blind). At each age, mononuclear cells were identified by indirect immunohistochemistry (IHC) using chicken marker-specific mouse monoclonal antibodies, biotinylated horse-antimouse IgG secondary antibody, and Vekta-stain Elite ABC reagents (streptavidin and biotin-conjugated peroxidase). Size-bar = 100 μm. Data are mean ± SEM; per age group, *n* = 6 and 3 for control and SL-NV, respectively; SL-IV: *n* = 0 at 1 week, *n* = 1 at 4 weeks, and *n* = 10 at 12 weeks. **(A–C)** Within an age group and across sample categories (control-NV, SL-NV, and SL-IV), mean levels of individual cell types without a common letter are different (*P* ≤ 0.05).

All SL chickens which exhibited signs of visual impairment also had vitiligo (Table 2). Microscopic evaluations of eye sections revealed normal morphology of choroidal melanocyte and RPE in all fully sighted chickens at 1 and 4 weeks of age, although for one 4-week SL-NV sample, there were signs of minor clumping/rounding of melanocytes and uneven sections of the columnar RPE. In the 12-week group, all SL-NV and controls exhibited normal melanocyte morphology in choroids with minor damage to the RPE cells. However, for all SL-IV eyes, the choroidal melanocyte score was greatly elevated compared to SL-NV and controls, with severe clumping of melanocytes/pigment and/or absence of pigment. Damage to the RPE in the eyes of partially sighted SL-IV birds was minor and not different from controls, whereas, in the eyes of blind SL-IV chickens, the RPE was severely damaged or nonexisting/visible (Figure 1A and Table 2).

**TABLE 2:** Choroidal melanocyte and retinal pigment epithelium (RPE) degeneration scores for eyes which were collected from age-matched controls and Smyth line chickens with and without vitiligo.

Age (weeks)	Line of chicken^1^	Number of birds	Vitiligo status^2^	Vision score^3^	Melanocyte score^4^	RPE score
1	Control	6	No-Vit	NV	1.00 ± 0.00	1.00 ± 0.00
1	SL	3	No-Vit	NV	1.00 ± 0.00	1.00 ± 0.00
4	Control	6	No-Vit	NV	1.00 ± 0.00	1.00 ± 0.00
4	SL	3	No-Vit	NV	1.25 ± 0.25	1.25 ± 0.25
12	Control	6	No-Vit	NV	1.00 ± 0.00 c	1.25 ± 0.20 b
12	SL	3	No-Vit	NV	1.00 ± 0.00 c	1.50 ± 0.50 ab
8 & 12^5^	SL	4 & 3	Vitiligo	IV Partial	2.25 ± 0.16 b	1.71 ± 0.18 ab
12	SL	3	Vitiligo	IV Blind	3.00 ± 0.00 a	2.50 ± 0.50 a

*^1^The control group consisted of three chickens from the parental Brown line and the vitiligo-resistant Light-brown Leghorn in each age group; SL = vitiligo-prone Smyth line of chicken.*

*^2^Birds either had vitiligo (Vitiligo) or did not exhibit pigmentation loss in growing feathers (No-Vit).*

*^3^Based on visual acuity tests (11), birds were categorized as having normal vision (NV) or impaired vision (IV); vision impairment was further categorized as partial or complete (blind) vision loss.*

*^4^Choroidal melanocyte and RPE damage scores were assigned using a scale from 1 (no damage) to 3 (extensive damage/loss).*

*^5^Data for eyes collected from four 8-week-old SL-IV chickens with partial vision and vitiligo are included in the 12-week SL-IV-partial sample group because statistical comparisons revealed no differences between the 8- and 12-week sample groups for any of the parameters examined. Data are mean ± SEM; (A–C): Within a column and age group, means without a common letter are different (P ≤ 0.05).*

The overall appearance of eyes in blind SL-IV birds was different from those of SL-NV. Blind SL-IV birds had swollen, vitiliginous eyelids, and sunken eyes with irregular-shaped pupils, pink sclera, and brown-red-colored iris instead of the yellowish iris with an outer black rim seen in SL-NV (Figure 1B).

Choroids from control and SL-NV chickens in the 1-, 4-, and 12-week sample groups were shown to have similarly low levels (1–2% area) of all mononuclear leukocyte populations examined [macrophages, γδ-TCR+−, CD4+−, and CD8+−T cells, and B cells (Bu-1+; IgM+)]. At 1 week, levels of MHC class II+ cells were low (1–2%) and not different between controls and SL-NV. However, at 4 and 12 weeks, SL-NV had 2- to 3-fold higher (*P* = 0.025 and 0.018, respectively) levels of MHC class II+ cells than controls (Figure 1C). Choroids in SL-IV eyes from the 12-week sample group had extensive (*P* ≤ 0.001) mononuclear cells infiltration, consisting predominantly of CD4+ and CD8+ T cells (11–12%, each), followed by γδ TCR+ T cells (6%), B cells (7%), and macrophages (9%). Nearly 20% of cells in the choroid were MHC class II+ in SL-IV. The one 4-week-old SL-IV chick exhibited similar mononuclear cell profiles and MHC class II-staining in choroids as the chickens in the 12-week SL-IV group (Figure 1C).

### Targeted Gene-Expression Analysis by Real-Time Reverse-Transcriptase PCR

**FIGURE 2:**
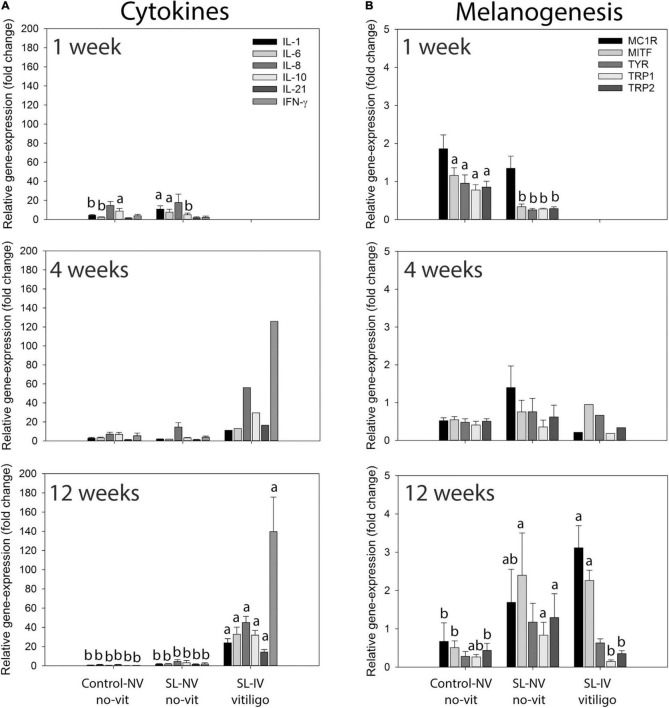
Expression of cytokine and melanogenesis-related genes in eye tissue from healthy controls and Smyth line (SL) chickens with and without vitiligo and visual impairment. **(A)** Relative mRNA levels of interleukin-1 (IL-1), IL-6, IL-8 (CXCL8), IL-10, IL-21, and interferon-γ (IFN-γ); **(B)** relative mRNA levels of melanocortin 1-receptor (MC1R), melanocyte-inducing transcription factor (MITF), tyrosinase (TYR), tyrosinase-related protein-1 (TRP1), and TRP2 (dopachrome tautomerase) in eyes from 1-, 4-, and 12-week-old controls, SL with normal (NV) and impaired vision (SL-IV; partial vision and blind). Relative mRNA expression of cytokines and melanogenesis-related genes in collected eyes was determined using qPCR. Data are mean ± SEM; for each age-group, *n* = 6 and 3 for control-NV and SL-NV, respectively; SL-IV: *n* = 0 at 1 week, *n* = 1 at 4 weeks, and *n* = 10 at 12 weeks. **(A,B)** Within an age group and across sample categories (control-NV, SL-NV, and SL-IV), mean mRNA expression levels of individual target genes without a common letter are different (*P* ≤ 0.05).

Cytokine mRNA-expression analysis (Figure 2A) of eye tissue from 1-week-old chicks revealed higher levels (fold change) of IL-1 (*P* = 0.039) and IL-6 (*P* = 0.042), and lower levels of IL-10 (*P* = 0.048), in SL-NV than controls, while levels of IL-8, IL-21, and INF-γ were similarly low in both groups. There were no differences in these cytokine mRNA levels between controls and SL-NV at 4 and 12 weeks. At 12 weeks, expression of all cytokines examined was higher (*P* ≤ 0.001) in SL-IV than in control and SL-NV eyes, with IFN-γ reaching the highest levels. A similar cytokine mRNA expression profile was observed in one 4-week-old SL-IV chick with partial vision (Figure 2A).

Relative expression of melanogenesis-related genes MC1R, MITF, tyrosinase, TRP1, and TRP2 varied with age group, line of chicken, visual acuity, and vitiligo status (Figure 2B). At 1 week of age, expression of MC1R was similar in eyes from controls and SL-NV. However, the relative expression of MITF, tyrosinase, TRP1, and TRP2 was lower (*P* ≤ 0.05) and below 1 in SL-NV than in controls. At 4 weeks, expression levels of melanogenesis-related target genes were not different between controls and SL-NV, although variability was greater in SL-NV samples. For the 12-week groups, MC1R expression was lower (*P* ≤ 0.05) in controls than in SL-IV, and intermediate in SL-NV. Expression of MITF was similarly elevated in SL-NV and SL-IV and higher (*P* ≤ 0.05) than in controls. There were no differences in tyrosinase expression levels between the three groups, although mean levels were highest (above 1) and most variable in SL-NV. Expression of TRP1 was higher (*P* ≤ 0.05) in SL-NV than in SL-IV samples, whereas TRP1 expression in controls was not different from either SL-NV or SL-IV. For TRP2 expression, levels in SL-NV were higher than in controls, with similarly low expression in controls and SL-IV (Figure 2B).

## Discussion

Studies in the 1980s on the then newly developed autoimmune vitiligo SL (aka DAM) chicken model described a loss of vision that only occurred in association with the cutaneous pigmentation loss in feathers. Moreover, histological evaluation of the autoimmune lesion in GF and eyes revealed many parallels in epidermal and choroidal melanocyte degeneration, including melanosome abnormalities and participation of mononuclear leukocytes (7, 11, 13, 22). Based on these studies, it was evident that the SL vitiligo model offered a unique opportunity to understand the mechanisms and processes underlying spontaneous autoimmune uveitis and its effects on the RPE and photoreceptors. In this study, conducted nearly 40 years later with the only remaining SL population, we not only confirmed the previously described mononuclear cell infiltration and melanocyte degenerative processes in eyes from SL chickens with vitiligo but also established the phenotype, cytokine expression, and relative proportions of infiltrating mononuclear cells in affected eyes.

Similar to the autoimmune activities in GF, T cells dominated the mononuclear cell infiltrate in choroids of vision-impaired SL chickens with similarly high levels (% area) of CD4+ helper and CD8+ cytotoxic T cells (11 and 12%, respectively), γδ T cells (6%), macrophages (9%), and B cells (7%). The same cell types were present at similar proportions in the GF-pulp during active vitiligo, although at lower levels (17). A major role of T cells in the spontaneous posterior uveitis and loss of choroidal melanocytes in SL chickens supports earlier reports that cyclosporine A treatment suppressed and delayed the onset of both the ocular and integumental autoimmune pathology (23). Moreover, as for active SLV, mRNA expression analysis of SL-IV eye tissue revealed a cytokine profile dominated by IFN-γ and accompanied by cytokines IL-1, IL-6, and IL-8, and also IL-10 and IL-21. The relative expression levels (fold change) of IFN-γ, IL-10, and IL-21, which were designated as the signature cytokines in SLV, were, however different in eyes and GF with active vitiligo (140, 15, and 30 in SL-IV eyes and 20, 37, and 38 in GF, respectively) (17). The discrepancy in leukocyte infiltration and cytokine expression levels between eye tissue and GF, may be explained by the tissues analyzed. Analyses of GF tissue included the melanocyte containing modified epidermis (barb ridge) which is only about 1/3 of the pulp epidermis and less than 5% of the entire epidermal and dermal portions of the pulp column (21). In eyes, analyses were focused on the location of the target cells, i.e., choroid for IHC and eye tissue devoid of vitreous and pecten for mRNA expression, respectively.

In this study, eyes were collected from different individuals at 1, 4, and 12 weeks of age, representing ages before SLV, the early phase of SLV before the visible loss of feather pigmentation, and active SLV, respectively (17). All SL-IV samples were collected within 3 weeks of SLV development, independent of age group (8 or 12 weeks) and extent of visual impairment (partial to blind). Moreover, mononuclear leukocyte profiles, MHC class II staining, and cytokine profiles were not different in SL-IV birds, whether partially sighted or blind. One notable difference was, however, severe damage to the RPE which was associated with extensive mononuclear cell presence in the eyes of SL-IV blind birds, whereas in partially sighted SL-IV birds, there was no or only minor damage to the RPE, and mononuclear leukocyte infiltration and melanocyte degeneration/loss was contained within the choroid. Smyth and collaborators reported similar observations, suggesting that the loss/damage of RPE in blind SL chickens may be secondary to the inflammatory autoimmune activities directed against choroidal melanocytes (11, 23, 12). The degree of damage to the RPE and retinal degeneration was found to be approximately proportional to the severity of the inflammatory reaction in the choroid in the current and previous studies (11, 12). Once the RPE is functionally damaged, the RPE-dependent photoreceptors cells degenerate as does the adjacent retina. As suggested by Fite et al. (12), the inflammatory reaction and buildup of pigment cell debris may interfere with the normal nutrient and waste transport function of the RPE to and from the photoreceptor cells, respectively, leading to retinal degeneration (12). Inherent defects in SL-RPE cells, e.g., reduced phagocytic activity, may also play a role in the autoimmune loss of these cells (24, 25).

Differences in immune activities were also observed between controls and SL-NV at all ages. MHC class II expression was higher in SL-NV than in controls at 4 and 12 weeks and numerically elevated at 1 week. MHC class II expression may be the first sign of loss of ocular immune privilege and indicative of future SL-IV development (26). As in SLV, MHC class II expression was localized to B cells, macrophages, endothelial cells of venules, and to melanocytes (15, 17). At 1 week, SL-NV also had higher expression of inflammatory cytokines IL-1 and IL-6 and lower levels of anti-inflammatory cytokine IL-10 than controls. This trend in pro- and anti-inflammatory cytokine expression was no longer evident at 4 and 12 weeks.

Melanogenesis in choroidal melanocytes and RPE is generally considered to be complete within the first weeks of life in mammalian and avian species (14, 27–29). Our data on normally sighted controls, especially at 4 and 12 weeks of age, agree with this concept, whereas data on SL-NV eyes, suggest aberrant melanogenic activity. Specifically, at 1-week, mRNA expression of MITF, TYR, TRP1, and TRP2 was downregulated, while at 12 weeks mRNA expression of these genes and MC1R were elevated in SL-NV compared to controls. In SL-IV, expression of MC1R and MITF also were higher than in controls but not different from SL-NV, whereas expression of TRP1 and TRP2 was lower than in SL-NV and not different from controls. Boissy et al. (14) reported elevated, postnatal tyrosinase activity in choroids, but not RPE, in SL adults compared to controls. In this study, TYR expression also was elevated in SL eyes at 12 weeks (juveniles), independent of vitiligo status. Further study is needed to define the dysregulation of melanogenesis in SL-NV and SL-IV eye tissue. Interestingly, melanogenic activity was observed in cultured human choroidal melanoma cells but not in choroidal melanocytes from healthy individuals (30), and human uveal melanocyte cell lines derived from human choroids and iris revealed that uveal melanocytes do not express MC1R (31).

In summary, the tight link between the development of uveitis, loss of choroidal melanocytes, and vision impairment, and the manifestation of spontaneous autoimmune vitiligo in SL chickens are maintained in the only remaining population of SL chickens. All chickens with vision impairment had vitiligo and the vision problems were detected within 3 weeks of SLV onset. Our data support the hypothesis that the autoimmune activities that drive the pathogenesis of spontaneous SL uveitis and vision impairment parallel those involved in cutaneous SLV. Specifically, as in active SLV, the loss of choroidal melanocytes is associated with an INF-γ polarized, Th1 cell-mediated immune response and aberrant melanocyte function. The immunopathogenesis in SL vision impairment resembles induced uveitis and human ocular diseases, especially Vogt–Koyanagi–Harada syndrome and sympathetic ophthalmia, both of which are autoimmune disorders associated with vitiligo (7, 32–34). Hence, the Smyth chicken model provides the opportunity to expand our understanding of spontaneous autoimmune pigmentation disorders, including autoimmune pigmentation loss associated with melanoma therapy (35), and develop effective treatment and prevention strategies.

## Data Availability Statement

The raw data supporting the conclusions of this article will be made available by the authors, without undue reservation.

## Ethics Statement

The animal study was reviewed and approved by the University of Arkansas IACUC.

## Author Contributions

GE: conceptualization, resources, project administration, and writing original draft. JS: animal experiments and IHC and histology analyses. WH and KB: gene-expression analyses. JS, WH, and GE: statistical analyses and funding acquisition. GE, JS, KB, and WH: review and editing. JS and GE: visualization. KB and GE: supervision. All authors contributed to the article and approved the submitted version.

## Conflict of Interest

The authors declare that the research was conducted in the absence of any commercial or financial relationships that could be construed as a potential conflict of interest.

## Publisher’s Note

All claims expressed in this article are solely those of the authors and do not necessarily represent those of their affiliated organizations, or those of the publisher, the editors and the reviewers. Any product that may be evaluated in this article, or claim that may be made by its manufacturer, is not guaranteed or endorsed by the publisher.
